# Evaluating private hospital performance before and during COVID-19 in China

**DOI:** 10.1097/MD.0000000000038327

**Published:** 2024-05-24

**Authors:** Xiaowen Wang, Jian Xu

**Affiliations:** a Financial Department, Qingdao Qingte Zhongji Axle Co., Ltd., Qingdao, China; b Department of Economics and Management, Qingdao Agricultural University, Qingdao, China.

**Keywords:** COVID-19, performance analysis, private hospital, profitability

## Abstract

The coronavirus disease 2019 (COVID-19) pandemic had a tremendous impact on the global medical system. The development of private hospitals is an important measure to deepen the reform of China’s medical and health system, and an important driving force to improve the effective supply of medical services. This study aims to compare the performance of China’s private hospitals before and during COVID-19 and determine the factors that affect hospital profitability between the 2 periods. Data are collected from 10 private listed hospitals from 2017 to 2022, and ratio analysis is used to measure hospital performance in 5 aspects, namely profitability, liquidity, leverage, activity (efficiency), and cost coverage. Multiple regression analysis is used to determine the influencing factors of hospital profitability. The results show a negative impact of COVID-19 on private hospital performance. Specifically, regardless of region, hospital profitability, liquidity, and cost coverage were reduced due to COVID-19, while hospital leverage was increased. COVID-19 had also an impact on hospital efficiency. In addition, before COVID-19, current ratio and cost coverage ratio were the determinants of hospital profitability, while only cost coverage ratio affected hospital profitability during the COVID-19 outbreak. We provide evidence that COVID-19 had an impact on China private hospitals, and the findings will aid private hospitals in improving their performance in the post-COVID-19 era.

## 1. Introduction

The coronavirus disease 2019 (COVID-19) pandemic has had significant impacts on global public health system, economy, and society since the beginning of 2020.^[1–3]^ It is no doubt that hospitals made great efforts to treat patients with COVID-19.^[4]^ However, the outbreak of COVID-19 led to a shortage of medical and healthcare resources and medical workers, and it also put pressure on the normal operation of hospitals.^[5,6]^ Therefore, it is important to focus on how hospitals operate to respond to COVID-19.

Private hospitals, as an important component of China medical system, play an important role in meeting the medical needs of the people.^[7]^ Compared with public hospitals, private hospitals are totally dependent on private capital.^[8]^ In addition, profit maximization is the main goal of private hospitals, while public hospitals need to protect the health of the people rather than profit pursuit.^[9]^ In China, private hospitals outnumber public counterparts, and they tend to face many challenges such as less access to government support, high tax burdens, and poor government regulation and oversight.^[8]^ In addition, their operational efficiency is low, and there are no economies of scale.^[10]^ What’s more, there was a decrease in private hospital profitability in the era of COVID-19.^[11]^ Therefore, ensuring the financial health and long-term development of private hospitals is beneficial to the stability of China medical system.^[12]^

Some studies have analyze the organizational performance before and during COVID-19 in companies,^[13–15]^ banks,^[16–18]^ and libraries,^[19]^ and very few studies have focused on the performance of medical institutions.^[20,21]^ For example, Nunes and Ferreira^[21]^ concluded that the efficiency of public hospitals dropped when COVID-19 started, and then recovered to prepandemic levels because of the actions taken by the Portuguese state. Caldas and Varela^[22]^ also confirmed a drop in Portuguese public hospitals’ performance during COVID-19. In addition, evaluating private hospital performance attracts less attention from scholars.^[9]^

In terms of profitability determinants in hospitals, Rosko et al^[23]^ pointed out that hospital efficiency, size, concentration of output, and system membership positively affect profitability in U.S. hospitals. Another study showed that the profitability of highest quality hospitals in the US is related to the case mix index and daily bed capacity.^[24]^ In a study by Cho and Hong,^[25]^ hospital type, the number of Medicaid patients, total hospital cost, and total bed size are influencing factors of hospital profitability. Rauscher and Wheeler^[26]^ explored the impact of working capital management on hospital profitability, and found a negative relationship between average collection period and average payment period and hospital profitability. Creixans-Tenas and Arimany-Serrat^[27]^ argued that the profitability of hospital companies is influenced by acceptable liquidity and indebtedness. In a word, little has been done on comprehensively analyzing the influential variables of private hospitals’ profitability, especially in emerging markets.

Performance management theory suggests that if companies want to maintain their competitive advantage in today market environment, they must improve their performance. Performance management can comprehensively reflect the organization operations and help efficiently achieve the goals, thus reducing the cost of companies. Based on this, the objective of this study is to analyze the performance of China private hospitals between the pre-pandemic and pandemic periods. Private hospitals listed on the Shanghai and Shenzhen stock exchanges are selected as the sample, and hospital performance is measured from 5 aspects, that is, profitability, liquidity, leverage, activity (efficiency), and cost coverage. In addition, we examine the determinants of hospital profitability before and during COVID-19 by using multiple regression analysis.

This study might contribute to the existing literature in 3 ways. First, based on financial indicators, we compare the changes in China private hospital performance before and during COVID-19 for the first time, which could enrich the literature on private hospital performance. Second, we evaluate factors that influence hospital profitability before and during COVID-19, and it might enhance the understanding of how private hospitals operate in times of crisis. Finally, the research results will aid private hospital managers in improving performance by making reasonable resource allocation strategies when facing crises.

## 2. Methods

### 2.1. Research sample

The sample of this study is 10 private hospitals listed on the Shanghai and Shenzhen stock exchanges during 2017 to 2022. These 10 hospitals are the representatives of Chinese private hospitals, and their development can better demonstrate the development achievements of private hospital industry and promote the healthy development of the entire medical system. We have excluded hospitals with missing information and hospitals delisted from the stock market. The COVID-19 broke out worldwide at the beginning of 2020.^[28]^ In order to analyze hospital performance changes, we set the period from 2017 to 2019 (before COVID-19) and the period from 2020 to 2022 (during COVID-19). The balanced panel data are collected from the CSMAR database and analyzed with the aid of Microsoft Excel and SPSS 22 software. In this study, the approval of IRB is not required.

### 2.2. Variables

Guided by Dong,^[29]^ Kim and Jeong-Kyo,^[30]^ and Jalilian et al,^[31]^ ratios in this study can be categorized into 5 aspects: profitability, liquidity, leverage, activity (efficiency), and cost coverage. Profitability ratios measure the hospital’s ability to generate profit,^[31]^ including return on assets (ROA), operating profit margin (OPM), and net profit margin (NPM). Liquidity ratios measure the hospital ability to meet its short-term financial obligations. The 3 liquidity ratios comprise current ratio (CUR), quick ratio (QUR), and cash ratio (CAR). Leverage ratios measure the ability of hospitals to fulfill their debts, which consists of ratio of total costs to total assets (TCTA), debt to asset ratio (DAR), and current debt ratio (CDR). Activity (efficiency) ratios measure the efficiency of hospitals in asset management, including receivables collection period (RCP), asset turnover ratio (ATR), inventory turnover ratio (ITR), and average inventory (INV). Cost coverage ratio measures the hospital ability to meet different sources of cost, with the introduction of operating cost coverage from operating income (OCOI). Table 1 presents the variable definition.

**Table 1:** Variable definition.

Variable	Indicator	Measurement
Profitability ratio	Return on assets (ROA)	Net income/total assets
	Operating profit margin (OPM)	Operating income/sales
	Net profit margin (NPM)	Net income/sales
Liquidity ratio	Current ratio (CUR)	Current assets/current liabilities
	Quick ratio (QUR)	(Current assets-inventory-prepaid expenses)/current liabilities
	Cash ratio (CAR)	Cash and cash equivalents/current liabilities
Leverage ratio	Ratio of total costs to total assets (TCTA)	Total costs/total assets
	Debt to asset ratio (DAR)	Total liabilities/total assets
	Current debt ratio (CDR)	Current liabilities/current assets
Activity (efficiency) ratio	Receivables collection period (RCP)	Average accounts receivable × 365/credit sales
	Asset turnover ratio (ATR)	Net revenue/average total assets
	Inventory turnover ratio (ITR)	Cost of goods sold/average inventory
	Average inventory (INV) (unit: million yuan)	(Inventory at the beginning of the period + inventory at the end of the period)/2
Cost coverage ratio	Operating costs coverage from operating income (OCOI)	Operating income/operating costs

## 3. Results

### 3.1. Descriptive statistics

The descriptive statistics are shown in Table 2. During the observed period, profitability ratios were negative, which means that COVID-19 had a harmful impact on total income of private hospitals. According to the China Health Statistics Yearbook 2021, there were approximately 23,500 nonpublic medical institutions in 2020 with an overall loss of about 130 billion yuan. In our sample, we find that 8 out of 10 private hospitals witnessed financial losses from 2017 to 2022. In 2021 and 2022, the losses of these hospitals were 2.81 billion yuan and 2.71 billion yuan, respectively, which suggests that most private hospitals were under huge financial pressure during COVID-19. In addition, cost coverage ratio was also negative.

**Table 2:** Descriptive statistics.

Variable	Indicator	Mean	Max	Min	Standard deviation
Profitability ratio	ROA	−0.0129	0.3070	−0.5087	0.1434
	OPM	−0.0488	1.4969	−2.6281	0.5091
	NPM	−0.0884	1.1000	−2.5985	0.4876
Liquidity ratio	CUR	1.3678	5.0899	0.2729	0.9782
	QUR	1.2674	4.9651	0.2359	0.9478
	CAR	0.6752	2.7842	0.0266	0.6533
Leverage ratio	TCTA	0.4336	0.9500	0.0853	0.1980
	DAR	0.4767	0.9901	0.0683	0.2381
	CDR	1.1251	3.6642	0.1965	0.7698
Activity (efficiency) ratio	RCP	65.6	180.0	5.7	44.3514
	ATR	0.4747	1.1150	0.0736	0.2476
	ITR	20.1125	78.9393	0.7330	18.6930
	INV	172.5048	743.2986	16.6578	197.0761
Cost coverage ratio	OCOI	−0.0443	1.9216	−5.5311	0.9088

ATR = asset turnover ratio, CAR = cash ratio, CDR = current debt ratio, CUR = current ratio, DAR = debt to asset ratio, INV = average inventory, ITR = inventory turnover ratio, NPM = net profit margin, OCOI = operating costs coverage from operating income, OPM = operating profit margin, QUR = quick ratio, RCP = receivables collection period, ROA = return on assets, TCTA = ratio of total costs to total assets.

### 3.2. Analysis results

Table 3 presents the percentage of changes in all variables during the 2 periods (2017–2019 and 2020–2022). Regarding profitability ratios, ROA, OPM, and NPM were negative in the COVID-19 era, and they had a dramatic decline. OPM was positive before COVID-19 and dropped more significantly during COVID-19, which reached -0.1397. As for liquidity ratios, all ratios were decreased considerably during COVID-19. CUR, QUR, and CAR had a change of approximately 10%. In terms of leverage ratios, TCTA, DAR, and CDR increased during COVID-19. The highest change was correlated with CDR, representing a change of about 35%. In activity (efficiency) ratios, RCP and ATR experienced a decrease during COVID-19, which suggests that private hospitals had weak short-term solvency and a high risk of bad debts after the COVID-19 breakout. By contrast, ITR rose from 17.2696 to 22.9554, and the increased ITR and the decreased INV suggest the strong liquidity of hospital inventories after COVID-19. Cost coverage ratio experienced a great decrease from 0.1239 to −0.2126.

**Table 3:** Comparison of hospital performance before and during COVID-19.

Variable	Indicator	Before COVID-19	During COVID-19	Changes (%)
Profitability ratio	ROA	−0.0006	−0.0252	4100.00
	OPM	0.0421	−0.1397	−431.83
	NPM	−0.0115	−0.1654	1338.26
Liquidity ratio	CUR	1.4686	1.2671	−13.72
	QUR	1.3381	1.1967	−10.57
	CAR	0.7199	0.6304	−12.43
Leverage ratio	TCTA	0.4284	0.4388	2.43
	DAR	0.4196	0.5338	27.22
	CDR	0.9590	1.2911	34.63
Activity (efficiency) ratio	RCP	69.8	61.4	−12.03
	ATR	0.4922	0.4572	−7.11
	ITR	17.2696	22.9554	32.92
	INV	206.8542	138.1554	−33.21
Cost coverage ratio	OCOI	0.1239	−0.2126	−271.59

ATR = asset turnover ratio, CAR = cash ratio, CDR = current debt ratio, CUR = current ratio, DAR = debt to asset ratio, INV = average inventory, ITR = inventory turnover ratio, NPM = net profit margin, OCOI = operating costs coverage from operating income, OPM = operating profit margin, QUR = quick ratio, RCP = receivables collection period, ROA = return on assets, TCTA = ratio of total costs to total assets.

Table 4 shows the trend of changes in profitability ratios before and during COVID-19. ROA, OPM, and NPM decreased significantly from 2018 to 2019, and rose again in the first year of COVID-19. During COVID-19 outbreak, 3 ratios fluctuated steadily.

**Table 4:** The trend of changes in all variables during 2017–2022.

Variable	Indicator	2017	2018	2019	2020	2021	2022
Profitability ratio	ROA	0.0483	0.0515	−0.1015	−0.0014	−0.0323	−0.0419
	OPM	0.1948	0.2212	−0.2897	−0.2251	−0.0921	−0.1019
	NPM	0.1550	0.1420	−0.3314	−0.2447	−0.1175	−0.1339
Liquidity ratio	CUR	1.5879	1.5106	1.3074	1.5634	1.1146	1.1233
	QUR	1.3863	1.4035	1.2245	1.4924	1.0554	1.0421
	CAR	0.8496	0.6166	0.6936	0.7248	0.5474	0.6192
Leverage ratio	TCTA	0.4087	0.4390	0.4376	0.4026	0.4427	0.4713
	DAR	0.3937	0.4033	0.4617	0.4954	0.5506	0.5555
	CDR	0.8236	0.8695	1.1841	1.2487	1.2199	1.4049
Activity (efficiency) ratio	RCP	75.6	64.0	69.9	68.9	64.0	51.2
	ATR	0.5370	0.4923	0.4474	0.4403	0.4767	0.4547
	ITR	17.2954	16.3941	18.1194	21.4846	25.4182	21.9634
	INV	245.7394	222.1841	152.6391	130.2562	133.6176	150.5926
Cost coverage ratio	OCOI	0.3745	0.3409	−0.3435	−0.4527	−0.0867	−0.0985

ATR = asset turnover ratio, CAR = cash ratio, CDR = current debt ratio, CUR = current ratio, DAR = debt to asset ratio, INV = average inventory, ITR = inventory turnover ratio, NPM = net profit margin, OCOI = operating costs coverage from operating income, OPM = operating profit margin, QUR = quick ratio, RCP = receivables collection period, ROA = return on assets, TCTA = ratio of total costs to total assets.

In this study, we also analyze hospital performance changes by region. China eastern regions include Beijing, Tianjin, Hebei, Shanghai, Jiangsu, Zhejiang, Fujian, Shandong, Guangdong, and Hainan, and other provinces are dedicated to central and western regions. Eastern regions have economic advantages, while central and western regions have resource advantages. Table 5 presents the comparison results. Before COVID-19, private hospitals in central and western regions had higher profitability, while they suffered poorer performance after COVID-19. However, private hospitals in eastern regions continued to suffer financial losses during the 2 periods. Liquidity ratios were decreased, while leverage ratios were increased during COVID-19 regardless of region. We find that private hospitals in central and western regions experienced greater fluctuations in profitability, liquidity, and leverage than those in eastern regions. It can be inferred that economic growth can mitigate the negative impact of COVID-19 to a certain extent. In activity (efficiency) ratio analysis, compared to before COVID-19, ATR and INV were decreased in the period of COVID-19, while ITR was increased during COVID-19 regardless of region. In cost coverage ratio analysis, regardless of region, OCOI was decreased greatly during COVID-19.

**Table 5:** Comparison of hospital performance before and during COVID-19 by region.

Variable	Indicator	Eastern regions			Central and western regions		
		Before COVID-19	During COVID-19	Changes (%)	Before COVID-19	During COVID-19	Changes (%)
Profitability ratio	ROA	−0.0085	−0.0308	262.35	0.0074	−0.0195	−363.51
	OPM	−0.0285	−0.0441	54.74	0.1126	−0.2354	−309.06
	NPM	−0.0744	−0.0685	−7.93	0.0515	−0.2622	−609.13
Liquidity ratio	CUR	1.7845	1.7828	−0.10	1.1527	0.7514	−34.81
	QUR	1.6309	1.7089	4.78	1.0453	0.6844	−34.53
	CAR	0.9396	0.8942	−4.83	0.5002	0.3667	−26.69
Leverage ratio	TCTA	0.4178	0.4599	10.08	0.4391	0.4178	−4.85
	DAR	0.3872	0.4478	15.65	0.4519	0.6198	37.15
	CDR	0.8007	0.8091	1.05	1.1174	1.7732	58.69
Activity (efficiency) ratio	RCP	58.4	62.4	6.85	81.2	60.4	−25.62
	ATR	0.5131	0.4714	−8.13	0.4713	0.4431	−5.98
	ITR	20.7960	30.8625	48.41	13.7432	15.0482	9.50
	INV	217.2754	98.7670	−54.54	196.4330	177.5439	−9.62
Cost coverage ratio	OCOI	0.0407	−0.0087	−121.38	0.2072	−0.4166	−301.06

ATR = asset turnover ratio, CAR = cash ratio, CDR = current debt ratio, CUR = current ratio, DAR = debt to asset ratio, INV = average inventory, ITR = inventory turnover ratio, NPM = net profit margin, OCOI = operating costs coverage from operating income, OPM = operating profit margin, QUR = quick ratio, RCP = receivables collection period, ROA = return on assets, TCTA = ratio of total costs to total assets.

We further examine the determinants of hospital profitability before and during COVID-19 with 3 profitability ratios (ROA, OPM, and NPM) being dependent variable. In order to avoid multi-collinearity, liquidity ratio includes CUR, and leverage ratio includes DAR. We find that all values of variance inflation factor (VIF) are <5. Models are specified in Table 6, and regression results are shown in Table 7. Before COVID-19, CUR had a negative impact on profitability ratios, while OCOI had a positive impact. During COVID-19, OCOI became the only determinant of hospital profitability. We notice that the impact of OCOI on hospital profitability before COVID-19 was higher than that during COVID-19. In addition, leverage and activity (efficiency) ratios had no significant impact on hospital profitability during the pre-COVID-19 and COVID-19 periods.

**Table 6:** Model specification.

No.	Model
1	ROA = β_0_ + β_1_CUR + β_2_DAR + β_3_RTR + β_4_ATR + β_5_ITR + β_6_INV + β_7_OCOI + ε
2	OPM = β_0_ + β_1_CUR + β_2_DAR + β_3_RTR + β_4_ATR + β_5_ITR + β_6_INV + β_7_OCOI + ε
3	NPM = β_0_ + β_1_CUR + β_2_DAR + β_3_RTR + β_4_ATR + β_5_ITR + β_6_INV + β_7_OCOI + ε

ATR = asset turnover ratio, CAR = cash ratio, CDR = current debt ratio, CUR = current ratio, DAR = debt to asset ratio, INV = average inventory, ITR = inventory turnover ratio, NPM = net profit margin, OCOI = operating costs coverage from operating income, β is the presumed parameter, ε is the term error, OPM = operating profit margin, QUR = quick ratio, RCP = receivables collection period, ROA = return on assets, TCTA = ratio of total costs to total assets.

**Table 7:** Regression results.

Variable	Before COVID-19			During COVID-19		
	Model (1)	Model (2)	Model (3)	Model (1)	Model (2)	Model (3)
Constant	0.099 (1.021)	0.080 (0.572)	0.011 (0.096)	−0.100 (−0.849)	−0.093 (−0.905)	−0.352** (−2.214)
CUR	−0.051** (−2.169)	−0.056 (−1.626)	−0.081*** (−2.967)	0.008 (0.288)	−0.004 (−0.157)	0.044 (1.165)
DAR	−0.390*** (−3.181)	−0.104 (−0.589)	−0.135 (−0.956)	−0.037 (−0.303)	−0.071 (−0.657)	0.124 (0.747)
RCP	0.000 (−0.091)	0.000 (−0.655)	0.000 (0.126)	0.000 (−0.692)	0.000 (0.566)	0.001 (1.045)
ATR	0.163** (2.311)	−0.037 (−0.362)	0.084 (1.034)	0.082 (0.663)	0.165 (1.532)	0.200 (1.199)
ITR	0.001 (1.091)	0.001 (0.835)	0.001 (0.442)	0.002* (1.945)	0.000 (0.373)	0.001 (0.730)
INV	0.000 (1.225)	0.000 (1.030)	0.000 (1.066)	0.000 (1.204)	0.000 (−0.223)	0.000 (−0.183)
OCOI	0.174*** (8.298)	0.682*** (22.545)	0.637*** (26.372)	0.024 (0.961)	0.476*** (21.727)	0.456*** (13.476)
F	21.156***	95.314***	129.729***	3.746***	176.816***	70.421***
Adj. *R*^2^	0.830	0.958	0.969	0.399	0.977	0.944

*t*-values are in parentheses.

ATR = asset turnover ratio, CAR = cash ratio, CDR = current debt ratio, CUR = current ratio, DAR = debt to asset ratio, INV = average inventory, ITR = inventory turnover ratio, NPM = net profit margin, OCOI = operating costs coverage from operating income, OPM = operating profit margin, QUR = quick ratio, RCP = receivables collection period, ROA = return on assets, TCTA = ratio of total costs to total assets.

* *P* < 0.10.

** *P* < 0.05.

*** *P* < 0.01.

## 4. Discussion

The aim of this study is to compare whether private hospital performance is affected by COVID-19. In addition, it also examines the factors influencing hospital profitability before and during COVID-19. The results of financial indicators are discussed as follows.

Our results reveal that 3 profitability ratios (ROA, OPM, and NPM) were negative during the COVID-19 outbreak. The main reason is that many private hospitals with insufficient manpower and resources were forced to shut down in order to prevent cross-infection during the epidemic, resulting in a significant decrease in the number of admissions and surgeries and revenues.^[32–34]^ This provides evidence that COVID-19 caused great damage to the hospital system, and it was also confirmed by Jalilian et al,^[31]^ King et al,^[35]^ Byun et al,^[36]^ and He et al.^[37]^ Similarly, Chen et al^[38]^ argued that the revenue of public hospitals dropped dramatically during COVID-19, but recovered after COVID-19. In U.S. hospitals, operating margins decreased during the first 6 quarters of the COVID-19 crisis.^[39]^

Compared to the pre-COVID-19 period, profitability ratios, liquidity ratios, and cost coverage ratios had a decrease regardless of region, which accords with the findings of Jalilian et al^[31]^ and Devi et al.^[40]^ Decreased liquidity ratios affect the decision-making of such hospitals. The reference criteria of CUR should be higher than 2, while the mean values of CUR during pre-COVID-19 and COVID-19 periods do not reach this criterion, suggesting that private hospitals were unable to pay their short-term debt. The mismatch between operating income growth and operating cost growth resulted in a negative OCOI during this pandemic. Bozdemir et al^[41]^ claimed that medicine, material, intervention, and examination costs increased during this crisis. For health plan operators, Marques et al^[20]^ found that profitability, expenses, cost variation, and financial results were reduced, while there was an increase in current liquidity after COVID-19.

Leverage ratios increased in the era of COVID-19 regardless of region. A CDR of > 1 indicates current liabilities are more than current assets. Higher leverage is a predictor of financial risks and bankruptcy probabilities,^[42,43]^ and it might be related to low investment.

In terms of activity (efficiency) ratio, RCP and ATR was decreased during the pandemic. In managing inventory, there was an increase in ITR and a decrease in INV during COVID-19. However, Jalilian et al^[31]^ found an increase in RCP and ATR in the era of COVID-19. The findings imply that the speed of cash collection in private hospitals was slowed down. This might due to the delayed payment of their customers. Increased ITR and decreased INV mean that these hospitals effectively managed their inventories during COVID-19. The findings of Kim and Jeong-Kyo^[30]^ also revealed that private hospitals in Korea tend to efficiently utilize assets to make profits.

Before COVID-19, CUR and cost coverage ratio were the determinants of hospital profitability, while only the latter affected hospital profitability in the background of COVID-19. It is worth noting that the relationship between CUR and profitability was negative before COVID-19, suggesting that private hospitals tend to have liquidity risks. However, according to Dong,^[29]^ profitability, leverage, liquidity, operating efficiency, and costs are the factors that influence hospital performance and quality of care during COVID-19. According to Vrontis et al,^[44]^ managerial capabilities do not directly affect financial performance of the healthcare sector during COVID-19.

## 5. Conclusions

This study aims to analyze the changes in private hospital performance in China before and during COVID-19 and determine the factors influencing hospital profitability during the 2 periods. Ratio analysis is used to measure hospital performance in 5 aspects, namely profitability, liquidity, leverage, activity (efficiency), and cost coverage. The findings reveal that, regardless of region, hospital profitability, liquidity, and cost coverage were reduced as a result of COVID-19, while hospital leverage was increased. Due to the limited access to government subsidies and the decrease in the number of inpatients caused by lockdowns, private hospitals faced immense financial pressure. COVID-19 had also an impact on hospital efficiency. In addition, cost coverage ratio determined hospital profitability before and during COVID-19. High expenses such as equipment, rent, and expert fees ultimately led to a loss-making situation for private hospitals.

The theoretical contributions of this study are as follows. Firstly, this study enriches the extant literature on private hospital management by analyzing the changes in China private hospital performance from 5 aspects before and during COVID-19 for the first time. Secondly, this study explores the determinants of hospital profitability before and during COVID-19, which can help private hospital managers have an in-depth understanding of performance improvement through reasonable resource allocation in times of crisis.

We provide some practical implications to help improve private hospital performance. First, private hospitals should strengthen the management of current assets and costs and control the level of financial leverage. Second, they need to develop a “specialized, refined, and new” business model through chain operation, public-private partnerships, and resource integration to achieve large-scale operations. Meanwhile, private hospitals should pay attention to gender and age diversity of employees as they can enhance performance and patient satisfaction.^[45]^ Finally, the government should implement preferential policies such as tax reduction, optimize financing policies, and encourage social capital to invest in private hospitals.

There are some potential limitations in this paper. First, in this study, the sample is limited to private hospitals, and future studies can analyze the difference of the impact of COVID-19 on both public and private hospitals. Second, we only focus on the financial indicators of hospitals, and future studies could take into consideration service performance in order to systematically measure hospital performance.

## Author contributions

**Conceptualization:** Xiaowen Wang, Jian Xu.

**Data curation:** Jian Xu.

**Formal analysis:** Jian Xu.

**Funding acquisition:** Jian Xu.

**Methodology:** Xiaowen Wang, Jian Xu.

**Project administration:** Jian Xu.

**Software:** Jian Xu.

**Writing – original draft:** Xiaowen Wang, Jian Xu.

**Writing – review & editing:** Jian Xu.
